# An assessment of medical students’ proficiency regarding the hazards of radiological examinations on the health of workers and patients: a cross-sectional study from Palestine

**DOI:** 10.1186/s12995-020-00287-8

**Published:** 2020-12-03

**Authors:** Ahmed Awadghanem, Mahmoud Sbaih, Mohammad Hasoon, Zaher Yassin, Ahmad M. Samara, Mosab Maree, Sa’ed H. Zyoud

**Affiliations:** 1 Department of Radiology, An-Najah National University Hospital, Nablus, 44839 Palestine; 2 Department of Medicine, College of Medicine and Health Sciences, An-Najah National University, Nablus, 44839 Palestine; 3 Department of Clinical and Community Pharmacy, Department of Pharmacy, College of Medicine and Health Sciences, An-Najah National University, Nablus, 44839 Palestine; 4 Poison Control and Drug Information Center (PCDIC), College of Medicine and Health Sciences, An-Najah National University, Nablus, 44839 Palestine; 5 Clinical Research Center, An-Najah National University Hospital, Nablus, 44839 Palestine

**Keywords:** Medical students, Radiological examinations, Radiation hazard, Palestine

## Abstract

**Background:**

The use of radiological examination is increasing worldwide. Since radiation exposure can result in many health hazards, medical professionals, as well as medical students, should possess adequate knowledge regarding radiation and its related hazards to protect themselves and the patients. Many studies have assessed medical students’ knowledge on this topic, but never in Palestine. In this study, we aimed to examine Palestinian medical students’ awareness and knowledge regarding radiological examination modalities and their risks on themselves and their patients.

**Methods:**

This was an observational, cross-sectional, population-based study, conducted to assess the awareness of radiation exposure and its risks among Palestinian medical students. An online questionnaire was implemented on medical students at An-Najah National University. A total knowledge score that ranged from 0 to 22 was calculated for each participant, with higher scores indicating better knowledge regarding radiation doses and the related hazards.

**Results:**

Two hundred eighty and seven students participated in our study, with a response rate of 71%. The average knowledge score of the participants was 10.97 ± 4.31 out of a maximum of 22 points. Male participants and participants in advanced study years achieved better knowledge scores (*p*-values were 0.034 and < 0.001, respectively). Perceived radiology knowledge was significantly associated with the actual knowledge score among the participants (*p*-value< 0.001). Receiving radiology lectures in fourth and fifth years significantly affected the participants’ knowledge score (*p*-values were < 0.001).

**Conclusions:**

We found a severe lack of knowledge regarding radiation doses and related risks among medical students. Therefore, we recommend that medical schools update and supplement their curriculum regarding knowledge on radiation.

## Background

The use of radiological examination is increasing worldwide [1]. The majority of patients being hospitalized nowadays will undergo one or more radiologic examination [2]. In general population, exposure to medical radiation is increasing. For instance, since 1993 the use of computed tomography (CT) scans in the United States had tripled reaching approximately 70 million scans per year [3]. Considering the association between radiation exposure and many health hazards, medical staff should be aware of relevant hazards and the means to protect themselves and the patients [4]. Increased risk of developing cancer is assumed proportional with increased radiation doses in the linear no-threshold (LNT) model [5].

Physicians are responsible for determining whether the patient has to undergo radiologic examination based on risk-benefit analysis [6]. In 2001, Brenner et al. found that lifetime cancer risk was quantitatively correlated with the number of pediatric CT scans [7]. Internationally, about a third of all magnetic resonance imaging (MRI) and CT imaging examinations are considered unwarranted [8, 9]. Previous studies have shown concerning results indicating that both healthcare professionals and trainees are not sufficiently familiar with radiological dosage and relevant hazards [10–17]. More attention has been directed to physicians’ knowledge regarding radiology, which has been frequently rating as inadequate [18]. These findings have prompted increased attention to improving healthcare professionals’ knowledge regarding radiological hazards [10–17]. Physicians’ knowledge, in particular, should be appropriately evaluated and defects should be traced back to their education at medical schools [19–21].

In Palestine, however, the situation regarding radiological knowledge among medical students has never been examined. Therefore, our aim in the current study was to examine knowledge regarding radiation doses and hazards associated with different diagnostic radiologic tests among Palestinian medical students, as well as their awareness regarding the appropriate use of these tests. The results of this study provide crucial information to guide decision-makers in their efforts to improve educational plans, as well as clinical guidelines for the optimal use of radiological examination, which will ultimately improve the outcomes of patient care without putting their health or that of the healthcare team in unnecessary jeopardy.

## Methods

### Study design

We conducted an observational study, cross-sectional, to assess the awareness regarding radiation exposure and its risks among Palestinian medical students.

### Study setting

This study was held at An-Najah National University (NNU) in Nablus, Palestine, between September 2019 and January 2020. Participants were enrolled in this study based on the defined inclusion and exclusion criteria.

### Study population and sampling technique

Our target population in this study was undergraduate medical students at NNU. We reached out to 404 students to enroll in this study. The participants were selected using the convenience sampling technique.

### Inclusion and exclusion criteria

In order to be included in the study, the subject had to be a registered student at NNU and had started his or her clinical training (4th, 5th, and 6th years). Both genders were eligible to participate in the study. Participants whose questionnaires were incompletely filled were excluded from the study if the missing data were significant**.** Those who did not consent to participate in the study were also excluded.

### Study tool

We distributed a questionnaire electronically via an online survey process among NNU medical students. Participation in the survey was voluntary and anonymous. Collection time (time the survey remained open) was 5 months. In order to increase participation in the study, there were two reminders sent to participants. The questionnaire contained multiple-choice questions on radiation doses and associated hazards.

The questionnaire items were in two parts: the first part contained items on socio-demographic characteristics (gender, clinical year, the confidence of knowledge, and radiology education) and the second part, which contained radiation knowledge items, was adapted from a previously used tool [10, 22–25] and supplemented by additional items regarding common radiological modalities. According to the opinions of the relevant experts in such types of studies, the questionnaire was slightly modified according to the needs of the sample population. Each question in the second part of the questionnaire has a single correct answer out of four to six options. One mark was given for each correct answer and zero marks for each incorrect or ‘I do not know’ responses. The overall knowledge score’ range was 0–22, with higher scores corresponding to better knowledge on radiation doses and related hazards.

### Statistical analysis

Data analysis was done by SPSS, version 21. All variables were expressed as frequencies and percentages. Means and standard deviations were also presented for continuous variables. Medians and interquartile ranges were also used for variables that did not follow the normal distribution, after testing for that using the Kolmogorov-Smirnov test. To test for differences between different groups, we used the Kruskal-Wallis test and Mann-Whitney test, as appropriate. Statistical significance was assumed at *p*-value < 0.05.

## Results

### Demographic and educational characteristics

Two hundred and eighty-seven (287) students participated in our study, accounting for a response rate of 71%. Overall, 59.2% of the participants were females. They were distributed in the fourth, fifth, and sixth study year with a roughly balanced ratio (30.7, 32.4, and 36.9%, respectively). About half of all participants (48.1%) rated their radiology knowledge as average.

Regarding lectures on radiation knowledge, the percentages of participants who received such lectures during the fourth, fifth, and sixth study year were 36.2, 50.5, and 0.7%, respectively. However, only a minority (7.3%) reported receiving lectures on radiation protection specifically. Table 1 summarizes the participants’ responses to characteristics items in full.

**Table 1:** Demographic and educational characteristics of the participants

Characteristic	Frequency (%); ***N*** = 287
**Gender**	
Male	117 (40.8)
Female	170 (59.2)
**Clinical year**	
4th year	88 (30.7)
5th year	93 (32.4)
6th year	106 (36.9)
**Self-perceived radiology knowledge compared to other subjects**	
Excellent	5 (1.7)
Good	83 (28.9)
Average	138 (48.1)
Poor	57 (19.9)
No knowledge	4 (1.4)
**Received radiology lectures as a part of the anatomy course**	
Yes	28 (9.8)
No	259 (90.2)
**Received radiology lectures as a part of a clinical skills course**	
Yes	137 (47.7)
No	150 (52.3)
**Received Radiology lectures during 4th clinical year**	
Yes	104 (36.2)
No	183 (63.8)
**Received Radiology lectures during 5th clinical year**	
Yes	145 (50.5)
No	142 (49.5)
**Received Radiology lectures during 6th clinical year**	
Yes	2 (0.7)
No	285 (99.3)
**Received education on protection from radiation**	
Yes	21 (7.3)
No	266 (92.7)

### The participants’ knowledge regarding the radiological examination and associated hazards

Table 2 summarizes the frequencies and percentages of participants who responded correctly to knowledge items. The average knowledge score of the participants was 10.97 ± 4.31 (out of 22 points maximum). Notably, most participants failed to estimate the radiation dose that results from different radiological modalities in relation to background radiation (for CXR) or compared to the dose received from one CXR (for all other modalities). Only items asking about which radiological modalities used X-ray and another item asking about the most susceptible group to radiation were answered correctly by more than half the participants.

**Table 2:** Frequencies and percentages of participants who answered correctly for each of the knowledge items

Knowledge item*	Frequency (%)
**1. Which of the following modalities do you think uses X-rays?**	
a. MRI (no)	234 (81.5)
b. Chest X-ray (yes)	282 (98.3)
c. Ultrasound (no)	251 (87.5)
d. CT (yes)	198 (69.0)
e. Conventional fluoroscopy (yes)	194 (67.6)
f. Mammography (yes)	181 (63.1)
g. Angiography (yes)	155 (54.0)
**2. In a chest X-ray, the radiation dose is the same as natural background radiation received in how long? (less than 1 week)**	28 (9.8)
**3. For each of the following modalities, the radiation dose is approximately the same as how many chest x-rays?**	
a. Ultrasound of abdomen (zero)	131 (45.6)
b. CT of the abdomen (300–1000)	63 (22.0)
c. MRI of the abdomen (zero)	114 (39.7)
d. Abdominal X-ray (20–50)	51 (17.8)
e. Barium swallow (20–50)	33 (11.5)
f. MRI of the spine (zero)	93 (32.4)
**4. Which of the following involves the highest radiation exposure for the patient? (plain film of the abdomen)**	82 (28.6)
**5. Which of the following groups is the most sensitive to radiation? (children)**	240 (83.6)
**6. Which organ is the least sensitive to radiation? (kidney)**	131 (45.6)
**7. Which of the following modalities is responsible for most of the radiation received by the general population? (CT)**	89 (31.0)
**8. For each of the following modalities, do you think it increases the lifetime risk of developing cancer?**	
a. MRI (definitely no)	103 (35.9)
b. Chest X-ray (definitely yes)	64 (22.3)
c. Ultrasound (definitely no)	150 (52.3)
d. CT (definitely yes)	109 (38.0)

* The correct answer is indicated between parentheses after each knowledge item

### Relationship of participants’ knowledge scores and their demographic and educational characteristics

Table 3 presents the findings of comparing knowledge scores between participants in different groups based on their characteristics. We found that male participants achieved a significantly higher knowledge score compared to female participants (*p*-value = 0.034). We also found a significant positive correlation between the clinical year participants were in and their knowledge scores (*p*-value< 0.001). Perceived radiology knowledge was significantly associated with the actual knowledge score among the participants (*p*-value< 0.001). Receiving radiology lectures in fourth and fifth years, but not in the sixth year, significantly affected the participants’ knowledge score (*p*-values were < 0.001 for lectures during the fourth and fifth year and 0.426 for the sixth year).

**Table 3:** Relationship between the participants’ demographic and educational characteristics and their knowledge scores

Characteristic	Knowledge score ^**a**^ Median [Q1-Q3]	***P***-value*
**Gender**		
Male	12.00 [8.00–15.00]	**0.034** ^**b**^
Female	10.25 [7.00–13.63]	
**Clinical year**		
4th year	8.25 [6.00–10.50]	**< 0.001** ^**c**^
5th year	10.50 [8.50–14.00]	
6th year	13.25 [10.00–16.00]	
**Self-perceived radiology knowledge compared to other subjects**		
Excellent	10.50 [8.50–11.75]	**< 0.001** ^**c**^
Good	11.50 [8.00–15.00]	
Average	12.00 [9.00–15.13]	
Poor	8.00 [5.25–10.00]	
No knowledge	5.25 [2.50–8.00]	
**Received radiology lectures as a part of the anatomy course**		
Yes	11.25 [8.25–15.38]	0.412 ^b^
No	10.50 [8.00–14.00]	
**Received radiology lectures as a part of a clinical skills course**		
Yes	11.50 [8.50–14.50]	0.110 ^b^
No	10.00 [7.50–14.00]	
**Received Radiology lectures during 4th clinical year**		
Yes	9.00 [6.50–13.00]	**< 0.001** ^**b**^
No	12.00 [9.00–15.00]	
**Received Radiology lectures during 5th clinical year**		
Yes	12.50 [9.50–15.50]	**< 0.001** ^**b**^
No	9.00 [6.00–13.00]	
**Received Radiology lectures during 6th clinical year**		
Yes	8.00 [3.00- NA]	0.426 ^b^
No	10.50 [8.00–14.25]	
**Received education on protection from radiation**		
Yes	12.00 [8.75–15.00]	0.249 ^b^
No	10.50 [8.00–14.00]	

*Q1-Q3* Quartile 3, Quartile 3, *NA* not available

* Significant p-values are in bold

^a^ The overall knowledge score’ range was 0–22, with higher scores corresponding to better knowledge on radiation doses and related hazards

^b^ Statistical significance of differences calculated using the Mann–Whitney U test

^c^ Statistical significance of differences calculated using the Kruskal–Wallis test

## Discussion

In this study, we examined, for the first time in Palestine, knowledge levels regarding radiation exposure and related hazards among medical students. Our findings indicate a serious knowledge gap, as evidenced by medical students’ mean radiation knowledge score (10.97 ± 4.31 out of a 22-point maximum).

The results of this study are in concordance with reports from the relevant literature, which indicated a gap in vital radiation knowledge among medical students. These studies are stemming from the need to prepare medical students for their future duties as physicians how should understand the risks and benefits of different radiological examination tools in order to optimally use them. So far, the results of such studies, including the current study, have been concerning. Numerous studies have been conducted in recent years among medical students and physicians for that our purpose [17, 18, 22, 23, 26–33]. Previous studies have shown concerning results indicating that both healthcare professionals and trainees are not sufficiently familiar with radiological dosage and relevant hazards [10–17].

Only a minority of the participating students demonstrated sufficient knowledge regarding the radiation dose in a single chest x-ray (CXR), a very common imaging modality. This is a particularly worrying finding considering that CXR knowledge is considered basic and essential knowledge for understanding risks associated with other imaging modalities in perspective.

In our sample, male students scored higher than female students on radiation knowledge. This finding may be caused by an uneven encouragement for male students to pursue radiology education and career paths that their female counterparts and further investigation in this area are required. The finding that medical students in more advanced study years achieved higher knowledge scores may point to a positive accumulation of knowledge throughout medical school, but considering the low average knowledge score regarding radiation, this effect is still insufficient for students to gain the appropriate level of relevant knowledge. These findings seem to be consistent with a report from Australia which found that 55.3% of medical students were females, but only 33.0% of trainees in radiology were females [34], and 26.5% of the radiologist workforce was female [35]. To encourage female students to select radiology as an appealing career option, more research needs to find challenges and solutions.

Another interesting finding in our study was that students who reported having excellent radiological knowledge achieved lower knowledge scores than those how rated their knowledge as average. This finding could point to a lack of recognition of the existing knowledge gap among the students. This is consistent with another study that reported a negative correlation between perceived knowledge and achieved knowledge score [17]. In contrast to other studies, knowledge related to MRI and US were noticeable good within our sample [32]. This could be due to the nature of the students’ curriculum and the topics it focuses on. On the other hand, knowledge related to CT scan radiation was inadequate. This is particularly worrying concerning that CT is considered a major source of radiation exposure being responsible for 70% of medical radiation dose [36].

### Strengths and limitations

A major limitation of this study was its cross-sectional design, which determined associations but could not address causality. Also, the convenience sampling method may have limited the generalizability of our results. Another limitation was the single-center setting of the study.

On the other hand, this was the first study of its kind in Palestine, and it provided important information regarding radiation awareness. Additionally, we used a very cost-efficient data collection method and, at the same time, included a large sample size, which increased the representation of our target population.

## Conclusions and recommendations

In conclusion, we found a severe lack of knowledge regarding radiation doses and related risks among medical students in Palestine. Such a low level of knowledge calls for a reconsideration of the current curriculum of medical education regarding radiation knowledge and its relevance. Our results also showed that medical students specifically underestimated radiation risks associated with CT scans, and demonstrated little knowledge about X-ray doses associated with radiological examination tools. We highly recommend redesigning certain courses and lectures in medical schools’ curriculum to include more information on radiation doses, associated risks, and radiation protection strategies. We also recommend using better tools for assessing students’ knowledge prior to participating in the medical field to avoid the overuse of ionizing radiation modalities. Finally, we recommend conducting multi-centric studies that assess radiation knowledge in order to explore this problem on a larger scale.

## Data Availability

The datasets used and/or analyzed in this study will be made available by the corresponding author upon reasonable request.

## Abbreviations

NNU: An-Najah National University
CXR: Chest X-ray
MRI: Magnetic resonance imaging
CT: Computed tomography
LNT: linear no-threshold
IRB: Institutional Review Board
